# Development of RP-HPLC Method for the Simultaneous Quantitation of Levamisole and Albendazole: Application to Assay Validation

**DOI:** 10.1155/2018/5746305

**Published:** 2018-07-02

**Authors:** S. Sowjanya, Ch. Devadasu

**Affiliations:** ^1^Department of Pharmaceutical Chemistry, Vishwa Bharathi College of Pharmaceutical Sciences, Perecherla, Guntur 522 009, Andhra Pradesh, India; ^2^Department of Pharmaceutical Analysis & Quality Assurance, Vignan Pharmacy College, Vadlamudi, Guntur 522 213, Andhra Pradesh, India

## Abstract

A reverse phase high-performance liquid chromatographic (RP-HPLC) method was developed and validated for simultaneous estimation of levamisole and albendazole in drug substance and in its combinational dosage form. The analysis was carried out using* Inertsil ODS *C_18_ (4.6 x 150 mm, 5 *μ*m) column, and the separation was carried out using a mobile phase containing a buffer of pH 3.5 and acetonitrile (70:30 v/v) pumped at a flow rate of 1.0 mL/min with variable wavelength UV-detection at 224 nm. Both the drugs were well resolved in the stationary phase and the retention times were 2.350 min and 4.055 for levamisole and albendazole, respectively. The method was validated and shown to be linear in the concentration range of 15-45*μ*g/ml and 40-120*μ*g/ml for levamisole and albendazole, respectively. The limit of detection (LOD) and limit of quantification (LOQ) were determined based on standard deviation of the y-intercept and the slope of the calibration curve. LOD and LOQ values were 2.08*μ*g/ml and 6.03*μ*g/ml for levamisole and 3.15*μ*g/ml and 10.40*μ*g/ml for albendazole, respectively. The accuracy of the method was assessed by adding known amount of standard solution (75 %, 100 %, and 125% of the sample concentration) to the preanalyzed sample solution of 100% concentration. All the samples were prepared and analyzed in triplicate. The percentage mean recovery by standard addition experiments of levamisole and albendazole is 99.66% and 98.73%, respectively.

## 1. Introduction

Levamisole hydrochloride salt is an anthelmintic and immunomodulator belonging to a class of synthetic imidazothiazole derivatives. It was discovered at Janssen Pharmaceutica in 1966. Levamisole has been used in humans to treat parasitic worm infections and has been studied in combination with other forms of chemotherapy for colon cancer, melanoma, and head and neck cancer. The drug was withdrawn from the US and Canadian markets in 2000 and 2003, respectively, due to the risk of serious side effects and the availability of more effective replacement medications. Levamisole is chemically (6S)-6-phenyl-2H,3H,5H,6H-imidazo[2,1-b][1,3]thiazole. It is indicated for adjuvant treatment in combination with fluorouracil after surgical resection in patients with Dukes' stage C colon cancer and also used to treat malignant melanoma and head/neck cancer. Levamisole was originally used as an antihelminthic to treat worm infestations in both humans and animals. Levamisole is official in Indian Pharmacopoeia[1], European Pharmacopoeia [2], British Pharmacopoeia[3], and United States Pharmacopoeia [4].

**Figure 1:**
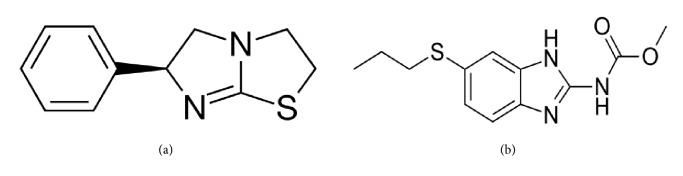
Chemical structures of (a) levamisole hydrochloride and (b) albendazole.

Albendazole is a member of the benzimidazole compounds used as a drug indicated for the treatment of a variety of worm infestations. Albendazole was first discovered at the SmithKline Animal Health Laboratories in 1972. It is a broad spectrum anthelmintic that is effective against roundworms, tapeworms, and flukes of domestic animals and humans. Albendazole, methyl N-[6-(propylsulfanyl)-1H-1,3-benzodiazol-2-yl]carbamate, is used for the treatment of parenchymal neurocysticercosis due to active lesions caused by larval forms of the pork tapeworm,* Taenia solium*, and for the treatment of cystic hydatid disease of the liver, lung, and peritoneum, caused by the larval form of the dog tapeworm,* Echinococcus granulosus*. Albendazole is official in the British Pharmacopoeia [5], the European Pharmacopoeia [6], and the United States Pharmacopoeia [7]. The chemical structures of the drug candidates selected for study were presented in Figure 1.

A thorough literature survey reveals that there were few analytical methods for estimation of albendazole, with other anthelmintic agents in various samples depending on the study employed. Some of the advanced instrumental methods have been developed earlier to determine the levamisole residues in cattle and swine livers by capillary gas chromatography-electron impact mass spectrometry by Stout SJ et al. [8], high-performance liquid chromatographic/gas chromatographic/tandem ion trap mass spectrometric determination of levamisole in milk by Chappell CG et al. [9], determination of levamisole in animal tissues using liquid chromatography-thermospray mass spectrometry by Cannavan A et al. [10], a liquid chromatographic-electrospray tandem mass spectrometric multiresidue method for anthelmintics in milk by Ruyck H et al. [11], quantitative analysis of levamisole in porcine tissues by high-performance liquid chromatography combined with atmospheric pressure chemical ionization mass spectrometry by Marc C et al. [12], liquid chromatographic determination of levamisole in animal plasma by Baere S et al. [13], determination of benzimidazoles and levamisole residues in milk by liquid chromatography-mass spectrometry by Piotr J et al. [14], a sensitive LC-MS/MS method for determination of levamisole in human plasma by Liping T et al. [15], development of a liquid chromatography-tandem mass spectrometry with pressurized liquid extraction method for the determination of benzimidazole residues in edible tissues by Chen D et al. [16], and determination of levamisole residue in animal livers by two liquid-liquid extraction steps, gas chromatography-mass spectrometry, by Xu J Xiao S et al. [17].

Some of the methods for determination of levamisole, along with some antihelminthics in various biological samples by HPLC, have been developed, including determination of the anthelmintic levamisole in plasma and gastrointestinal fluids by high-performance liquid chromatography by Marriner S et al. [18], determination of levamisole by HPLC in plasma samples in the presence of heparin and pentobarbital by Garcia JJ et al. [19], determination of levamisole and thiabendazole in meat by de Bukanski BW et al. [20], quantitation of levamisole in plasma using high-performance liquid chromatography by Vandamme TF et al. [21], solid-phase extraction and HPLC determination of levamisole hydrochloride in sheep plasma by Du Preez JL et al. [22], determination of levamisole in animal tissues using liquid chromatography by E. Dreassi et al. [23], quantitative chromatographic determination of several benzimidazole anthelmintic molecules in parasite material by Mottier L et al. [24], liquid chromatographic method with ultraviolet absorbance detection for measurement of levamisole in chicken tissues, eggs, and plasma done by El-Kholy H et al. [25], quantitative determination of albendazole metabolites in sheep spermatozoa and seminal plasma by Batzias GC et al. [26], a rapid HPLC method for analysis of ricobendazole and albendazole sulfone in sheep plasma by Wu Z et al. [27], HPLC assay of levamisole and abamectin in sheep plasma for application to pharmacokinetic studies by Sari P et al. [28], determination of levamisole in sheep muscle tissue by Tyrpenou AE et al. [29], and investigation of the persistence of levamisole and oxyclozanide in milk and fate in cheese by M. Whelan et al. [30]. Determination of levamisole with other anthelmintic agents in pharmaceuticals including veterinary preparations by HPLC includes high-performance liquid chromatography method for the analysis of several anthelmintics in veterinary formulations by E. C. Van-Tonder et al. [31], simultaneous determination of levamisole hydrochloride and mebendazole in tablets by R. Raman et al. [32], separation and determination of the process related impurities of mebendazole, fenbendazole, and albendazole in bulk drugs by Gomes AR et al. [33], simultaneous determination of levamisole and abamectin in liquid formulations by P. Sari et al. [34], simultaneous estimation of levamisole, mebendazole, and albendazole in pharmaceutical products by Kullai Reddy Ulavapalli et al. [35], method for determination of levamisole in bulk and dosage form by Ravisankar P et al. [36], enantioseparation of tetramisole by capillary electrophoresis and high-performance liquid chromatography and application of these techniques to enantiomeric purity determination of a veterinary drug formulation of L-levamisole done by B. Chankvetadze et al. [37], UPLC method for the determination of albendazole residues pharmaceutical manufacturing equipment surfaces by R. S. Chandan et al. [38], and HPTLC method for simultaneous estimation of levamisole hydrochloride and oxyclozanide in its bulk and pharmaceutical dosage form by Patel MB et al. [39]. Some of the GC methods for estimation of levamisole [40, 41] and determination of albendazole and its metabolites [42] in cheese [43] and in milk [44] have been developed. Few spectrophotometric methods have been developed, including the quantitative determination of albendazole with Chloramine-T and acid dyes by Basavaiah K et al. [45], ion-pair complex method by El-Didamony AM et al. [46], UV spectrophotometric method [47], derivative spectrophotometry method for determination of levamisole hydrochloride tablets by L. Liang et al. [48], extractive spectrophotometric method by Johnson Misquith et al. [49], mixture containing levamisole and triclabendazole in veterinary tablets by E. Dinc et al. [50], and estimation of mebendazole and levamisole hydrochloride in pharmaceutical formulations by Umang Shah et al. [51]. Some of the miscellaneous methods such as linear titrations for various samples have been developed [52–57].

## 2. Materials and Methods

Levamisole and albendazole gift samples were obtained from Chandra labs, Pvt. Ltd., Hyderabad. All chemicals and solvents were of analytical reagent grade. Acetonitrile and water (HPLC grade) were obtained from Merck Pvt. Ltd., Mumbai. Quantitative HPLC was performed on a high pressure gradient high-performance liquid chromatograph (Shimadzu HPLC, Class VP series) with two pumps, manual injector with loop volume of 20 *μ*L (Rheodyne), programmable variable wavelength UV detector, and a reversed phase column (Inertsil column, C_18_ (150 x 4.6 ID) 5*μ*m). The output signal was monitored and integrated using “Spincotech” software. A Systronics double beam UV-visible spectrophotometer 2203 with 1 cm matched quartz cells was used for all spectral and absorbance measurements and solutions were prepared in double distilled water.

### 2.1. Preparation of Mobile Phase

About 30 parts by volume of phosphate buffer were mixed with 70 parts by volume of acetonitrile and the resulting solution was filtered through a nylon membrane filter (pore size is 0.22 *μ*m). The mobile phase was degassed through ultrasonication.

### 2.2. Preparation of Standard Stock Solution of Levamisole

50 mg of levamisole was weighed, transferred into 500 ml volumetric flask, dissolved in methanol, and the resulting solution was diluted with methanol up to the mark. From the solution thus obtained, we carefully transferred 1 ml into a 10 ml volumetric flask and the volume was made up to the mark with methanol.

### 2.3. Preparation of Standard Stock Solution of Albendazole

50mg of albendazole was weighed into 500ml volumetric flask, dissolved in methanol, and then diluted up to the mark with methanol; 10*μ*g /ml of solution was prepared by diluting 1ml to 10ml with methanol.

### 2.4. Preparation of Mixed Standard Solution

Weigh accurately 150 mg of levamisole and 400 mg of albendazole in 100 ml of volumetric flask, dissolve them in 10ml of mobile phase, and make up the volume with mobile phase. From above stock solution 100*μ*g/ml of levamisole and 4*μ*g/ml of albendazole are prepared by diluting 1ml to 10ml with mobile phase. This solution is used for recording chromatogram.

### 2.5. Preparation of Sample Solution

Five tablets (each containing 150 mg of levamisole and 400 mg of albendazole) were weighed, taken into a mortar, crushed to fine powder, and uniformly mixed. Tablet stock solutions of levamisole (30*μ*g/ml) and albendazole 80*μ*g/ml) were prepared by dissolving weight equivalent to 150 mg of levamisole and 400 mg of albendazole dissolved in sufficient mobile phase. After that the solution was filtered using 0.45-micron syringe filter, sonicated for 5 min, and diluted to 100ml with mobile phase. Further dilutions were prepared in 5 replicates of 30*μ*g/ml of levamisole and 400*μ*g/ml of albendazole was made by adding 0.3ml and 0.8ml of stock solution to 10 ml of mobile phase.

### 2.6. Recommended Chromatographic Procedure for Assay

20 *μ*L of the standard solution was injected into the chromatographic system and chromatogram was recorded. 20 *μ*L of the standard solution was injected five times into the chromatographic system, chromatograms were recorded, and peak areas were measured. 20 *μ*L of the sample solution was injected two times into the chromatographic system, chromatograms were recorded, and peak areas were measured. The amount of the drug present in the formulation was computed by the following formula:

(1) $$\begin{array}{c} Amount=\frac{A_{T1}}{A_{S1}}\times \frac{D_{S1}}{D_{T1}}\times \frac{P1}{100}AW \end{array}$$

where

 
A_T1_ is the average area counts of levamisole peak in chromatogram of sample solution,
 
A_S1_ is the average area counts of levamisole peak in chromatogram of standard solution,
 
D_S1_ is the dilution factor for the standard solution,
 
D_T1_ is the dilution factor for the sample solution,
  P_1_ is the percentage potency of levamisole working standard used (as is basis),
  AW is the average weight of tablet.

## 3. Results and Discussions

### 3.1. Method Development and Optimisation

**Figure 2:**
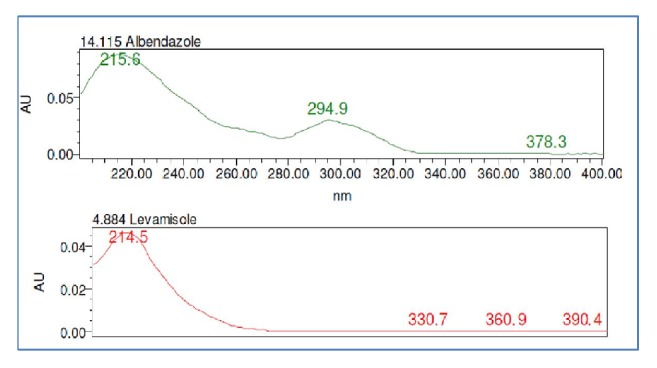
Individual UV spectrum of levamisole and albendazole.

The present study was carried out to develop a simple, sensitive, precise, accurate, rapid, and economical HPLC method for the quantification of levamisole and albendazole present in its formulation. Preliminary trails were conducted with these solvents to find out a suitable mobile phase composition where all the analytes were resolved with good system suitability. Mixture of phosphate buffer pH 3.5 and acetonitrile (30:70 v/v) was selected as mobile phase to enhance compatibility of the sample with the mobile phase and to avoid baseline disturbances. About 20*μ*g/ml of standard solution in methanol was scanned in the region between 200 and 400 nm. In simultaneous estimation of two drugs, isobestic wavelength is used. Isobestic point is the wavelength where the molar absorptivity is the same for two substances that are interconvertible. Therefore, this wavelength is used to estimate both drugs accurately. The isobestic point was found to be 224 nm for levamisole and albendazole; therefore, 224 nm was selected as detection wavelength. The UV absorption spectrum showing isobestic point of the two drug molecules was given in Figure 2, and the optimised chromatographic conditions were presented in Table 1.

### 3.2. Method Validation

**Figure 3:**
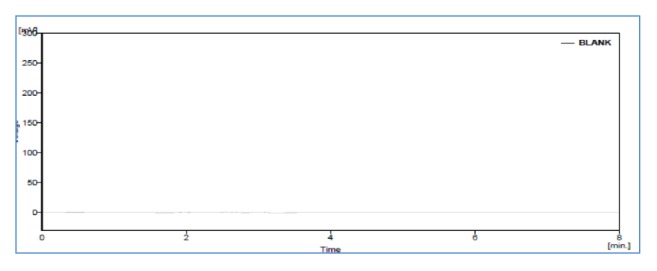
Blank chromatogram for specificity by using mobile phase.

The proposed method was validated as per the guidelines of ICH Q2R1. The assay method developed was subjected to validation by performing specificity, linearity, limit of detection, limit of quantification, precision, accuracy, and robustness. Specificity of the method was proved by demonstrating no excipients interference at the retention time of both the drugs in chromatogram of assay sample. The results of specificity were given in Figure 3.

**Figure 4:**
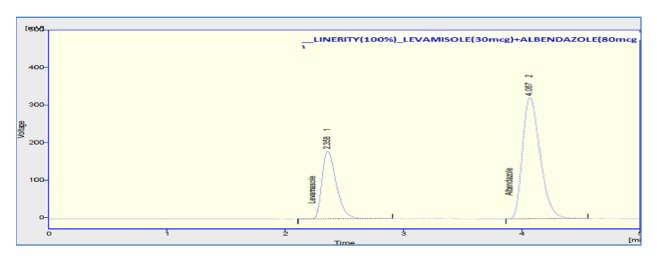
Chromatogram of levamisole and albendazole preparation-3.

**Figure 5:**
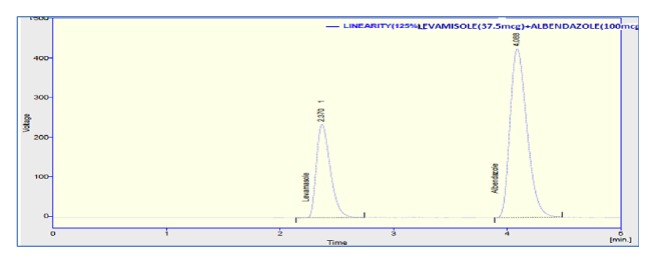
Chromatogram of levamisole and albendazole preparation-4.

**Figure 6:**
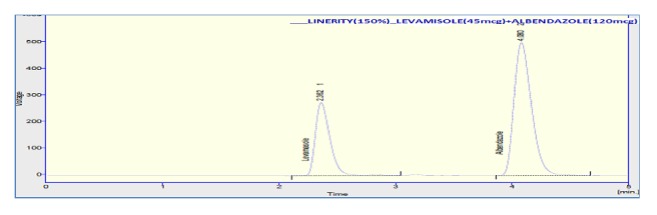
Chromatogram of levamisole and albendazole for preparation-5.

**Figure 7:**
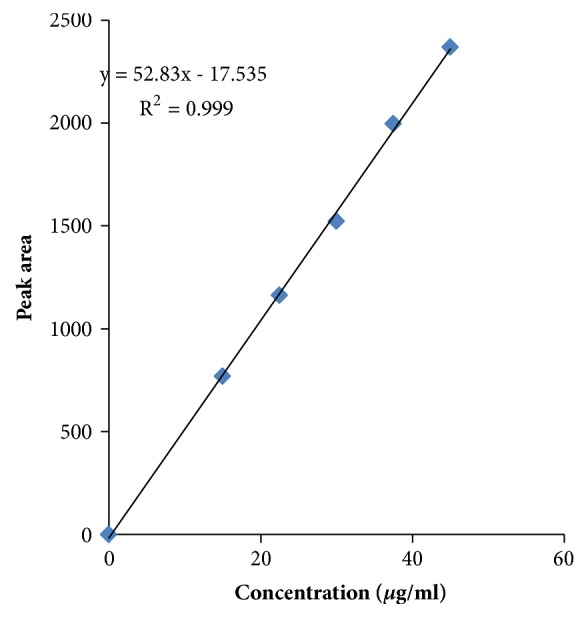
Calibration curve of levamisole.

**Figure 8:**
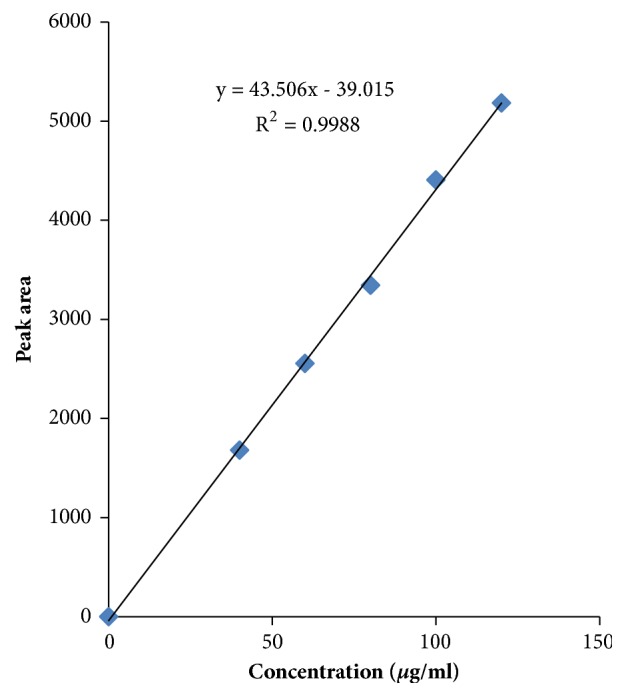
Calibration curve of albendazole.

Linearity graph displayed good linearity over the concentration range examined. The polynomial regression for the linearity plot showed good linear relationship with coefficient of correlation. The relationship between the concentration of the drugs and their area was linear in the range examined (15-45*μ*g/ml and 40-120*μ*g/ml for levamisole and albendazole, respectively). Since all points lie in a straight line and the correlation coefficients were well within limits. The correlation coefficients were found to be 0.998 and 0.999 obtained between concentration and area of standard preparations of levamisole and albendazole, respectively. The chromatograms concerned with linearity testing were given (for the two given concentrations only) in Figures 4–6. The results of linearity were presented in Table 2 and the corresponding calibration curves were given in Figures 7 and 8 for levamisole and albendazole, respectively. The limit of detection (LOD) and limit of quantification (LOQ) were determined based on standard deviation of the y-intercept and the slope of the calibration curve. LOD and LOQ values were 2.08*μ*g/ml and 6.03*μ*g/ml for levamisole and 3.15*μ*g/ml and 10.40*μ*g/ml for albendazole, respectively.

**Figure 9:**
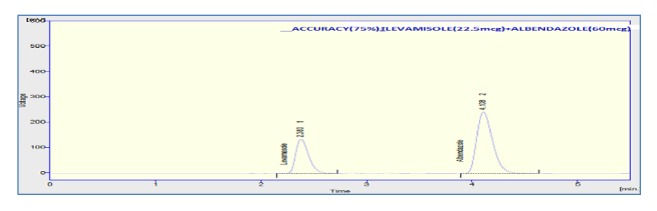
Chromatogram of 75% recovery.

**Figure 10:**
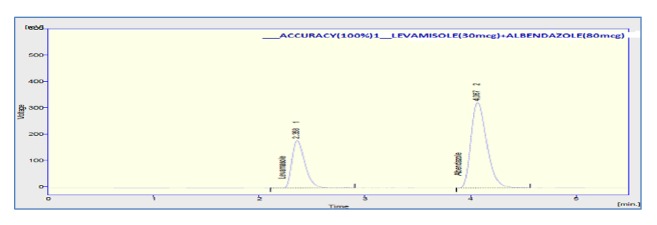
Chromatogram of 100% recovery.

**Figure 11:**
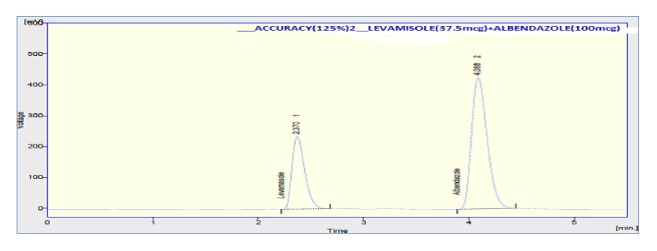
Chromatogram of 125% recovery.

**Figure 12:**
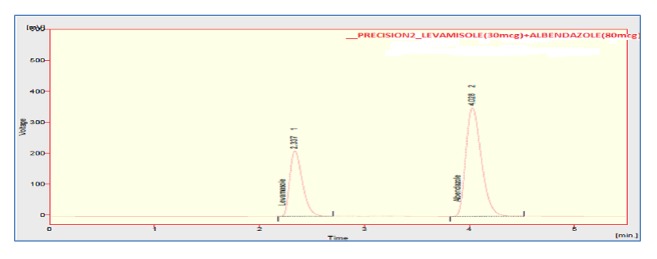
Chromatogram of precision injection 3.

**Figure 13:**
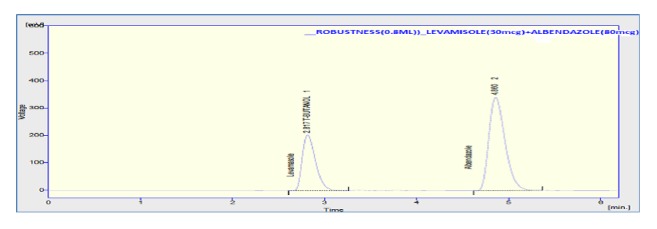
Chromatogram of levamisole and albendazole robustness (0.8 ml/min).

The accuracy of the method was assessed by adding known amount of standard solution (75 %, 100 %, and 125 % of the sample concentration) to the preanalyzed sample solution of 100% concentration. All the samples were prepared and analyzed in triplicate. The percentage mean recovery by standard addition experiments of levamisole and albendazole is 101.33 % and 98.73 %, respectively. High recovery values indicated proposed method for assay was highly accurate. The detailed results concerning the method accuracy were given in Figures 9–11 and Table 3. System precision, method precision, and intermediate precision were performed for the assay to evaluate precision of the method. % RSD of method precision was found to be** 0.46** and** 0.22** for levamisole and albendazole, respectively. Lower % RSD of precisions indicated that the method is precise. The results of precision are presented in Figure 12 and Table 4. The robustness of the method was evaluated to determine the capacity of the intended method to remain unaffected by changing organic phase composition of mobile phase, flow rate, and wavelength of detection. The developed method was found to be robust as these changes did not show significant effect on theoretical plates and tailing of the two drug candidates in assay and system suitability. Chromatogram showing the study of robustness is given in Figure 13, and the results of robustness and ruggedness are shown in Tables 5 and 6.

## 4. Conclusion

A simple and selective LC method is described for the determination of levamisole and albendazole dosage forms. Chromatographic separation was achieved on a C_18_ column using mobile phase consisting of a mixture of 20 Mm phosphate buffer (KH_2_PO_4_) pH and 3.5 acetonitrile (30:70 v/v), with detection of 224 nm. Linearity was observed in the range 15-45*μ*g /ml for levamisole (r^2^ =0.9975) and 40-120*μ*g /ml for albendazole (r^2^ =0.9994), as the amount of drugs estimated by the proposed methods was in good agreement with the label claim. The proposed methods were validated. The accuracy of the methods was assessed by recovery studies at three different levels. Recovery experiments indicated the absence of interference from commonly encountered pharmaceutical additives. The method was found to be precise as indicated by the repeatability analysis, showing % RSD less than 2. All statistical data proves validity of the methods and can be used for routine analysis of pharmaceutical dosage form. From the above experimental results and parameters, it was concluded that this newly developed method for the simultaneous estimation of levamisole and albendazole was found to be simple, precise, and accurate; high resolution and shorter retention time make this method more acceptable, and it can be effectively applied for routine analysis in quality control department in industries.

## Figures and Tables

**Table 1:** Optimized chromatographic conditions.

Mobile phase	Mixed Phosphate buffer (KH_2_PO_4_+K_2_HPO_4_): Acetonitrile 30:70
pH	3.5
Column	INERTSIL column, C18 (150x4.6 ID) 5*μ*m
Flow rate	1.0 ml/min
Column temperature	Room temperature (20-25°C)
Sample temperature	Room temperature (20-25°C)
Wavelength	224nm
Injection volume	20 *μ*l
Run time	10 min
Retention time	About 2.350min for Levamisole and 4.055min for Albendazole

**Table 2:** Linearity of levamisole and albendazole.

**S. No.**	**Levamisole**		**Albendazole**	
	**Concentration ** **(** ***μ*** **g/ml )**	**Area**	**Concentration ** **(** ***μ*** **g/ml )**	**Area**
1	15	768.60	40	1679.72
2	22.5	1163.20	60	2554.72
3	30	1522.81	80	3344.73
4	37.5	1996.49	100	4406.95
5	45	2368.14	120	5182.27

**Table 3:** Results of accuracy by recovery studies.

Recovery level	Amount added	Area	Amount Recovered	% Recovery	Average % recovery
		Levamisole			
**75 **%	22.5	1163.20	22.8	101.33	100.31
	22.5	1156.99			
	22.5	1158.28			
**100 **%	30	1522.81	29.82	99.41	
	30	1509.63			
	30	1515.34			
**125 **%	37.5	1996.49	37.57	100.19	
	37.5	1986.18			
	37.5	1990.97			
		Albendazole			
**75 **%	60	2554.72	59.34	98.90	98.73
	60	2533.53			
	60	2548.31			
**100 **%	80	3344.73	78.70	98.37	
	80	3342.78			
	80	3342.22			
**125 **%	100	4406.95	98.92	98.92	
	100	4394.54			
	100	4427.63			

**Table 4:** Results of intra-assay precision studies of levamisole and albendazole.

**Trail no.**	Rt	**Levamisole**			**Albendazole**	
		Area	Amount (mg)	Rt	Area	Amount (mg)
1	2.357	1768.18	151.37	4.060	3628.24	402.02
2	2.337	1767.59	151.25	4.028	3600.32	401.19
3	2.337	1757.73	149.89	4.028	3595.94	399.45
4	2.357	1772.34	151.87	4.060	3625.39	401.52
5	2.342	1769.77	151.68	4.035	3605.63	401.05
6	2.3460	1767.12	151.15	4.042	3611.10	401.76
		% RSD	**0.46**		%RSD	**0.22**

**Table 5:** Result of robustness study.

Parameter	**Levamisole**		**Albendazole**	
	**Retention ** **time(min)**	**Tailing factor**	**Retention ** **time(min)**	**Tailing factor**
**Flow**				
0.8ml/min	2.817	1.585	4.860	1.585
1.0ml/min	2.355	1.571	4.062	1.571
1.2ml/min	2.022	1.574	3.487	1.574
**Wavelength**				
222nm	2.367	1.571	4.080	1.571
224nm	2.355	1.571	4.062	1.571
226nm	2.367	1.535	4.082	1.535

**Table 6:** Results for ruggedness.

**Levamisole**	%**Assay**	**Albendazole**	%**Assay**
Analyst 01	97.99	Analyst 01	99.96
Analyst 02	98.37	Analyst 02	97.59
%RSD	**0.27**	%RSD	**1.69**

## Data Availability

Supporting data of the study is available from the corresponding author and may be given upon request.
